# Long-term healthcare use and costs in patients with stable coronary artery disease: a population-based cohort using linked health records (CALIBER)

**DOI:** 10.1093/ehjqcco/qcw003

**Published:** 2016-01-20

**Authors:** Simon Walker, Miqdad Asaria, Andrea Manca, Stephen Palmer, Chris P. Gale, Anoop Dinesh Shah, Keith R. Abrams, Michael Crowther, Adam Timmis, Harry Hemingway, Mark Sculpher

**Affiliations:** 1 Centre for Health Economics, University of York, York YO10 5DD, UK; 2 Faculty of Medicine and Health, Leeds Institute of Cardiovascular and Metabolic Medicine, University of Leeds, Leeds LS2 9JT, UK; 3 Farr Institute of Health Informatics Research, UCL Institute of Health Informatics, University College London, London WC1E 6BT, UK; 4 Department of Health Sciences, Centre for Biostatistics & Genetic Epidemiology, Leicester LE1 7RH, UK; 5 Department of Health Sciences, University of Leicester, Leicester LE1 7RH, UK; 6 NIHR Biomedical Research Unit, Barts and the London NHS Trust, London E1 2AD, UK

**Keywords:** Stable coronary artery disease, Electronic health records, Costs, Resource use

## Abstract

**Aims:**

To examine long-term healthcare utilization and costs of patients with stable coronary artery disease (SCAD).

**Methods and results:**

Linked cohort study of 94 966 patients with SCAD in England, 1 January 2001 to 31 March 2010, identified from primary care, secondary care, disease, and death registries. Resource use and costs, and cost predictors by time and 5-year cardiovascular disease (CVD) risk profile were estimated using generalized linear models. Coronary heart disease hospitalizations were 20.5% in the first year and 66% in the year following a non-fatal (myocardial infarction, ischaemic or haemorrhagic stroke) event. Mean healthcare costs were £3133 per patient in the first year and £10 377 in the year following a non-fatal event. First-year predictors of cost included sex (mean cost £549 lower in females), SCAD diagnosis (non-ST-elevation myocardial infarction cost £656 more than stable angina), and co-morbidities (heart failure cost £657 more per patient). Compared with lower risk patients (5-year CVD risk 3.5%), those of higher risk (5-year CVD risk 44.2%) had higher 5-year costs (£23 393 vs. £9335) and lower lifetime costs (£43 020 vs. £116 888).

**Conclusion:**

Patients with SCAD incur substantial healthcare utilization and costs, which varies and may be predicted by 5-year CVD risk profile. Higher risk patients have higher initial but lower lifetime costs than lower risk patients as a result of shorter life expectancy. Improved cardiovascular survivorship among an ageing CVD population is likely to require stratified care in anticipation of the burgeoning demand.

## Introduction

Improved survival coupled with a decline in the incidence of acute myocardial infarction (AMI)^1,2^ has dramatically changed the pattern of healthcare use over recent years.^3,4^ Nowadays, patients with stable coronary artery disease (SCAD), including patients with stable angina and those who have become stable after acute coronary syndrome (ACS),^5,6^ are older and living longer and so make greater use of healthcare resources. Patients with SCAD might be considered to have ‘fallen off the radar’ of clinical interest: no longer in cardiac rehabilitation [mainly offered to those immediately after AMI or coronary artery bypass grafting (CABG)], discharged from ongoing specialist care, and with suboptimal drug compliance, adherence, and persistence.^7^ Such patients, however, vary widely in their risk of subsequent AMI or coronary death (∼10-fold, between top and bottom deciles of risk),^8^ which will clearly have differential resource implications.

While previous studies of resource use and cost have taken as a starting point AMI,^9,10^ there is a paucity of information about the contemporary use and associated costs of healthcare beyond the initial hospital stay. In addition, existing studies in the area have been limited in a number of ways. First, as a result of the nature of their samples, they are not population based and do not reflect contemporary and routine clinical practice.^11^ Second, they use overly simplistic models, typically restricting their analysis to a subset of SCAD index events and not evaluating how the pattern of healthcare resource use changes following a first post-SCAD myocardial infarction (MI) or stroke.^9,10^ Third, no study has evaluated resource utilization and costs according to baseline cardiovascular risk, despite the importance of this information in improving decision-making and ensuring more efficient use of limited healthcare resources. Fourth, longer term and particularly lifetime implications of SCAD have not been quantified.

These knowledge gaps have a number of important ramifications. They create uncertainty for those who need to forecast future care needs, restrain the research and development of new technologies and treatments for SCAD, and limit informed clinical decision-making. To address these limitations, our study aimed to (i) determine healthcare utilization and the associated costs in the first year with SCAD and in the year following a first non-fatal event (i.e. AMI, ischaemic or haemorrhagic stroke), (ii) study predictors of costs in the first year of SCAD, and (iii) estimate the 5-year and lifetime costs among patients at low and high risk of subsequent events and coronary heart disease (CHD) death.

## Methods

### Data set and patient population

The ClinicAl research using Linked Bespoke studies and Electronic Records (CALIBER) e-health database was the data resource for this study. CALIBER links patient records from four different data sources: Clinical Practice Research Database (CPRD), Myocardial Ischaemia National Audit Project (MINAP) registry, Hospital Episodes Statistics (HES), and the Office for National Statistics. The data of CPRD were used to obtain heart rate measurements, demographic variables, and other risk factors. Primary care practices that provide valuable data to CPRD and cover ∼4% of UK population are representative in terms of demographic parameters such as gender, age, and ethnicity^12,13^ and overall mortality^14^ and have been validated for epidemiological research. A description of the CALIBER approach has been presented elsewhere.^15^ Classification algorithms combining Read, International Classification of Disease 10 (ICD-10), drug, and procedure codes to define risk factors and endpoints are available at http://www.caliberresearch.org/portal/.

Eligible patients were those with a diagnosis of stable angina, patients with a diagnosis of ACS within the study period (unstable angina or AMI), or those with a diagnosis of CHD in which there is no further specification of whether it is angina or MI (other CHD). Study start date was calculated from the date of diagnosis of stable angina or other CHD or from 6 months after an ACS or coronary intervention. The period of 6 months was chosen to differentiate long-term prognosis from the high-risk period that typically follows an ACS or revascularization. Diagnoses were identified in CPRD, HES, or MINAP records according to definitions given in the CALIBER data manual.^15^ Patients were only eligible for the study during the period they were actively registered at a CPRD practice that was collecting up-to-standard data (according to CPRD measures of data quality and completeness), with follow-up ending if a patient transferred out of a CPRD practice. Full details of the cohort are available elsewhere.^8^

### Healthcare utilization

Healthcare utilization extracted from the data set included primary care consultations, pharmaceutical prescriptions, inpatient stays, and diagnostic tests. Primary care consultations included all contacts between the patient and healthcare professionals captured in the CPRD data set. Prescription data were available from the CPRD data set and distinguished between drugs that were cardiovascular disease (CVD)-related and those that were not. Inpatient stays extracted from HES were based on Health Resource Group (HRG) codes and defined as CHD, CVD (including CHD and broader HRGs), or non-CVD related based on ICD-10 codes. Diagnostic tests as recorded in the primary care data set but not outpatient consultations were also extracted, the latter being due to the absence of outpatient HES data linkage.

### Costs

All costs were calculated from the perspective of the UK National Health Service (NHS) in pound sterling based on 2011/12 prices. Costs were calculated by combining healthcare utilization data from CALIBER with associated unit costs that were taken from published UK sources.^16–18^ For hospitalizations, costs are calculated based on finished consultant episodes in HES. Costs are presented in terms of total healthcare costs (all costs incurred), CHD costs (all CHD-related hospitalization costs, CVD-related drugs, and all primary care and diagnostic costs), and CVD costs (all CVD-related hospitalization costs, CVD-related drugs, and all primary care and diagnostic costs).

### Analytical methods

Estimates of healthcare utilization and costs were calculated for the first year in the SCAD cohort and the first year following a non-fatal event during the follow-up period (AMI, ischaemic or haemorrhagic stroke) with results reported as means and standard deviations, with medians and interquartile ranges reported in the appendices. Observations that were right censored for any reason other than mortality (i.e. those for which the data are incomplete for the year of interest, either first year with SCAD or first year following an event, but the reason for incompleteness was not death) were excluded from the analysis.

A generalized linear model with a log link and gamma distribution was used to estimate the impact of baseline covariates on costs in the first year in the SCAD cohort to account for the non-linear impact of covariates and the right skewness in cost data. Covariates were based on those used by Rapsomaniki *et al*.,^8^ which developed a prognostic model for SCAD patients, on the assumption that predictors of costs were likely linked to prognostic indicators. The covariates included the baseline diagnosis for entry to SCAD, co-morbidities, age, gender, smoking status, and biomarkers. Models were fitted on five multiply-imputed data sets and estimates combined using Rubin's rules.^19^ The impact of covariates has been transformed back onto the natural scale to allow for ease of interpretation.

Panel data methods with time invariant covariates were used to estimate patient costs over each 90-day period. Also estimated were the impact of events (non-fatal AMI, ischaemic and haemorrhagic stroke, CVD- and non-CVD-related death) on the costs in the period of the event and subsequent periods if the event was non-fatal. These costs were then combined with a state transition Markov model to estimate costs over a longer period. The model estimated the probabilities of, and mean times to, the first events of non-fatal AMI, ischaemic or haemorrhagic stroke, CVD- and non-CVD-related death, and subsequent CVD or non-CVD death following a non-fatal first event. These estimates were conditional on baseline covariates and were inferred from patient-level costs, covariates, survival, and events experienced. Full details of this model are available elsewhere with a brief description given in the appendix.^20^ For the purpose of this article, the results of costs over time are presented for patients based on average covariate patterns for the 5-year risk deciles of a cardiovascular event. Discounted costs are also presented using a discount rate of 3.5% per annum.^21^

## Results

### Cohort

In total, 94 966 patients were identified who met the inclusion criteria, of which 44% were female. The mean age of men and women included were 67 and 72 years, respectively. For the primary SCAD diagnosis, 47.4% of patients had stable angina, 13.5% unstable angina, 6.7% ST-elevation MI (STEMI), 9.7% non-STEMI (NSTEMI), and 22.6% had CHD not otherwise specified. Full details of the cohort can be found in Appendix *Table A1*.

**Figure 1:**
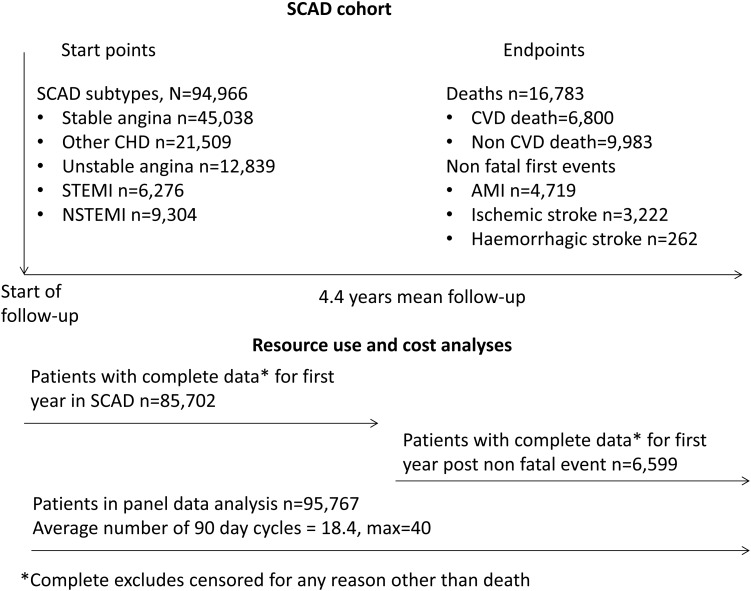
Stable coronary artery disease cohort.

*Figure 1* summarizes the SCAD cohort and the patient numbers used for each analysis.

### Healthcare utilization

*Table 1* reports healthcare utilization in the first year in the SCAD cohort and in the first year following a non-fatal event during the follow-up period (AMI, ischaemic or haemorrhagic stroke). In the first year in the SCAD cohort, 20.5% of patients (*n* = 17 532) were hospitalized for CVD, and those who were hospitalized had a mean 1.9 stays (spells) in hospital with a mean length of stay of 4.6 days. In the year following a non-fatal event during follow-up, 66% of patients (*n* = 4354) were hospitalized for CHD. These patients spent a mean of 2.2 stays in hospital with a mean length of stay of 6.5 days. In the first year in SCAD, patients had a mean of 10.8 primary care appointments, and this increased to 13.7 in the first year following a non-fatal event. In the first year of SCAD, 88.2% of patients were taking cardiovascular medication, which decreased to 83.6% in the year following a non-fatal event. In the first year of SCAD, 5.7% of patients had a revascularization procedure, increasing to 13.5% of patients in the first year following a non-fatal event.

**Table 1:** Healthcare utilization in first year in the stable coronary artery disease cohort and first year following a non-fatal event during follow-up (myocardial infarction, ischaemic or haemorrhagic stroke)

	Resource use in first year (*n*= 85 702)	Resource in first year following an event (*n* = 6599)
	Mean (SD)	Mean (SD)
Hospitalizations		
Hospitalized (%)	37.6 (0.484)	83.5 (0.371)
Hospitalized for CVD (%)	27 (0.444)	80.2 (0.399)
Hospitalized for CHD (%)	20.5 (0.403)	66 (0.474)
Inpatient stays	0.875 (4.421)	2.364 (6.425)
Inpatient stays for CVD	0.503 (2.545)	1.931 (5.284)
Inpatient stays for CHD	0.343 (1.321)	1.434 (3.315)
With any hospitalization	*n* = 32 242	*n* = 5512
Inpatient stays	2.325 (6.971)	2.83 (6.935)
Length of stay	6.717 (17.583)	14.857 (20.349)
With any hospitalization for CVD	*n* = 23 160	*n* = 5291
Inpatient stays for CVD	1.862 (4.631)	2.408 (5.803)
Length of stay	7.516 (12.757)	15.541 (20.939)
With any hospitalization for CHD	*n* = 17 532	*n* = 4354
Inpatient stays for CHD	1.674 (2.51)	2.174 (3.88)
Length of stay	4.579 (7.617)	6.515 (10.857)
Revascularizations		
Any revascularization (%)	5.7 (0.232)	13.5 (0.341)
PCI (%)	3 (0.17)	9.1 (0.287)
CABG (%)	2.9 (0.169)	5 (0.218)
Primary care consultations	10.768 (10.857)	13.671 (15.407)
Drugs		
Patients on any drugs (%)	91.3 (0.281)	85.1 (0.356)
Patients on CVD drugs (%)	88.2 (0.322)	83.6 (0.37)
Patients on anticoagulants (%)	8.7 (0.282)	14.7 (0.354)
Patients on ACEi or ARB (%)	47.7 (0.499)	61.5 (0.487)
Patients on anti-platelets (%)	65.6 (0.475)	76.3 (0.425)
Patients on β-blockers (%)	46 (0.498)	50.1 (0.5)
Patients on calcium channel blockers (%)	31.5 (0.464)	32.6 (0.469)

### Costs

*Table 2* reports costs for hospitalizations, primary care appointments, diagnostic tests, and drugs in patients in the first year in the SCAD cohort, and in the first year following a non-fatal event during follow-up (MI, ischaemic or haemorrhagic stroke). The mean total healthcare costs in the first year in the SCAD cohort were £3133 per patient, of which 56.8% (£1780) and 70.2% (£2199) were related to CHD and CVD, respectively. This estimate increased to £10 377 per patient in the year following a non-fatal event during follow-up, of which 66.2% (£6869) and 85.9% (£8916) were related to CHD and CVD, respectively. The majority of healthcare costs were driven by hospitalizations (64.4% in the first year in the SCAD cohort and 84.2% in the year following a non-fatal event during follow-up).

**Table 2:** Costs in first year in the stable coronary artery disease cohort and in first year following an event during follow-up (myocardial infarction, ischaemic or haemorrhagic stroke)

	Costs in first year (*n* = 85 702)	Costs in first year following an event (*n* = 6599)
	Mean (SD)	Mean (SD)
Total costs		
Total cost (£)	3133 (6101)	10 377 (12 260)
Total CVD cost (£)	2199 (4632)	8916 (10 930)
Total CHD costs (£)	1780 (3686)	6869 (9467)
Hospitalizations		
Inpatient costs (£)	2018 (5632)	8744 (11 554)
Inpatient CVD costs (£)	1487 (4493)	7957 (10 796)
Inpatient CHD costs (£)	1067 (3548)	5910 (9338)
Primary care costs (£)	466 (463)	589 (629)
Diagnostic test costs (£)	141 (232)	228 (311)
Drugs		
All drug costs (£)	508 (1548)	816 (3135)
CVD drug costs (£)	105 (113)	142 (175)

### Cost predictors in the first year in the stable coronary artery disease cohort

**Figure 2:**
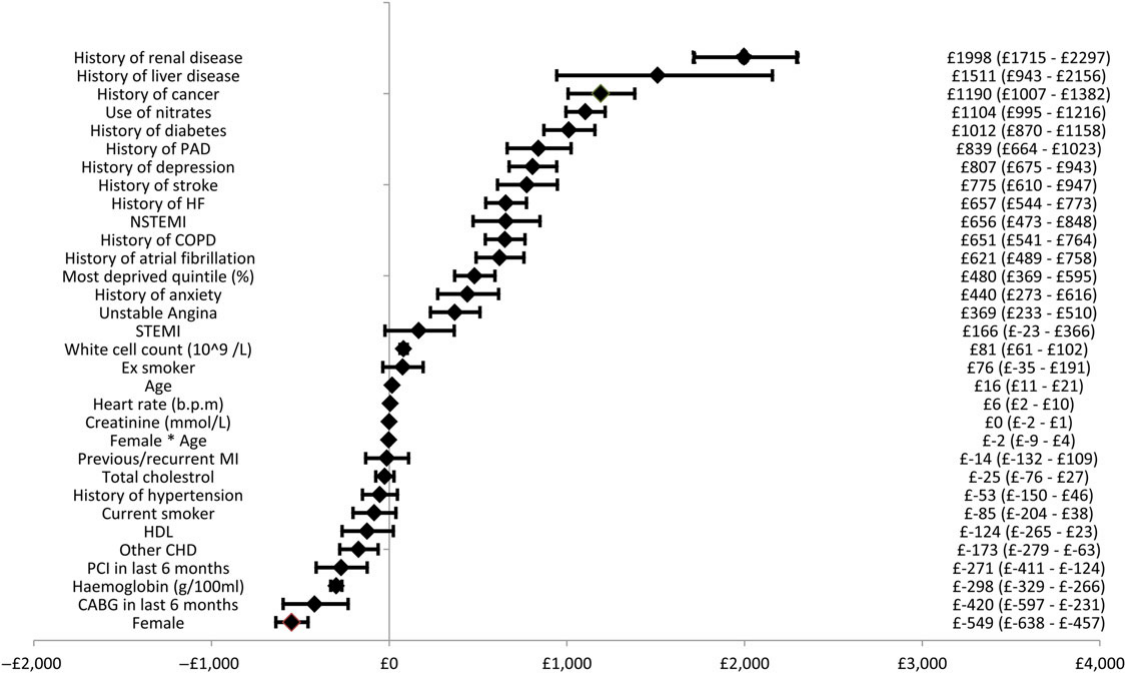
Forest plot of incremental costs associated with covariates.

**Figure A1:**
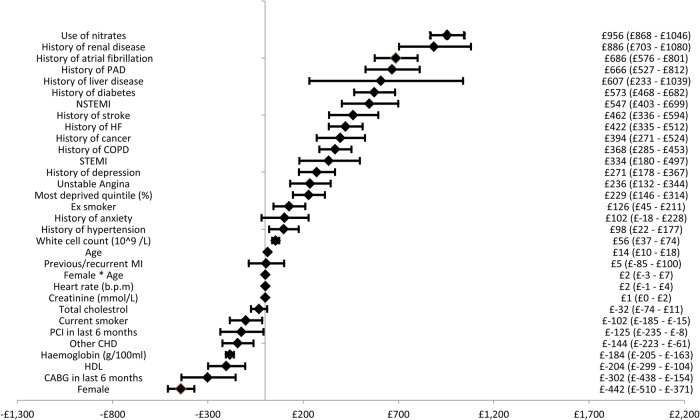
Forest plot of cardiovascular disease costs.

**Figure A2:**
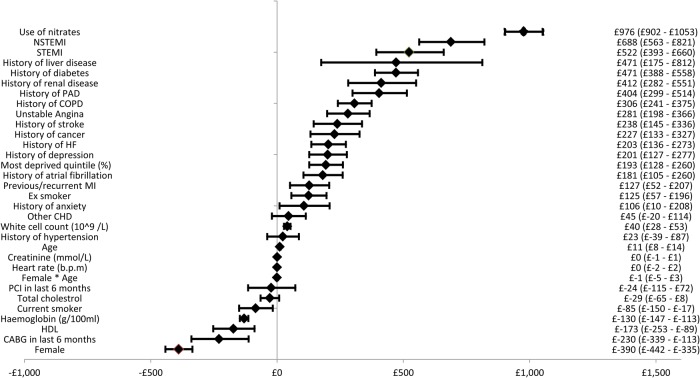
Forest plot of coronary heart disease costs.

*Figure 2* presents the results of the regression analysis showing the incremental costs associated with different covariates and the associated 95% confidence intervals (CI) for the first year in the SCAD cohort. Non-CVD-related co-morbidities had the largest impact on costs, with a history of renal disease associated with the largest increment of £1998 per patient (95% CI £1715–£2297). A history of heart failure resulted in an additional cost of £802 per patient (95% CI £683–£920). Of the baseline diagnoses for entry to the SCAD cohort, NSTEMI had the largest impact on cost with those patients with NSTEMI costing an additional £656 per patient (95% CI £473–£848) when compared with those with stable angina. Females were significantly less costly than males, being female was associated with a cost decrement of £549 per patient (95% CI −£638 to −£457). *Figures A1* and *A2* in the appendix present the same results for CVD- and CHD-related costs, respectively.

### Estimated 90-day period and event costs

*Tables A4*, *A5*, and *A6* in the appendix provide estimates of total healthcare costs, CVD-related costs, and CHD-related costs, respectively, for a 90-day period based on a range of baseline characteristics. The tables also show the incremental costs in the period of an event and in subsequent periods for non-fatal events. For example, the background total healthcare costs for a male, mean age 69 years, with no co-morbidities would be £341 in the first 90 days, increasing by £10 for each subsequent 90-day period. If the patient had a non-fatal AMI, he would incur an incremental cost (on top of the normal period cost of £372) of £5028 in the 90 days following the AMI with the incremental costs decreasing in subsequent trimesters until 360 days after which there is an incremental cost of £521 per 90 days suggesting significant ongoing lifetime costs of events. Also of note, the incremental costs in the period of death from CVD- and non-CVD-related causes were £2008 and £2240, respectively.

### Estimated costs over time for stable coronary artery disease

*Table 3* presents estimates of 5-year and lifetime costs (total and CVD related, undiscounted and discounted) of the representative patients for each risk group. The covariate profiles used are shown in Appendix *Table A7*.

**Table 3:** Mean 5-year and lifetime costs for stable coronary artery disease patient population by cardiovascular risk decile

Results	Risk group									
	1	2	3	4	5	6	7	8	9	10
5-year risk^a^ (%)	3.46	5.43	6.95	8.53	10.36	12.57	15.64	20.07	27.23	44.18
Average age (years)	52	59	62	65	68	70	73	76	80	84
Life expectancy (years)	26.81	19.62	17.34	15.63	14.26	13.03	11.92	10.48	8.52	5.51
Total 5-year costs (£)	9335	11 200	12 308	13 512	14 644	15 930	17 660	19 609	21 617	23 391
Discounted total 5-year costs^b^ (£)	8495	10 204	11 221	12 327	13 368	14 554	16 153	17 963	19 853	21 620
Total 5-year CVD costs (£)	5306	6959	7941	8954	9874	10 904	12 242	13 742	15 380	17 050
Discounted total 5-year CVD costs^b^ (£)	4823	6338	7238	8168	9014	9962	11 197	12 589	14 126	15 759
Total 5-year CHD costs (£)	4172	5543	6354	7153	7867	8583	9449	10 385	11 376	12 392
Discounted total 5-year CHD costs^b^ (£)	3801	5057	5801	6534	7191	7850	8651	9521	10 455	11 459
Total lifetime costs (£)	116 888	81 490	73 057	68 102	64 521	62 034	61 435	59 446	54 345	43 020
Discounted total lifetime costs^b^	62 210	50 864	48 046	46 535	45 429	44 785	45 283	44 903	42 436	35 549
Total lifetime CVD costs (£)	71 943	52 034	47 681	45 251	43 438	42 266	42 301	41 366	38 410	31 199
Discounted total lifetime CVD costs^b^ (£)	37 857	32 331	31 288	30 896	30 584	30 531	31 211	31 281	30 024	25 801
Total lifetime CHD costs (£)	46 921	36 069	33 892	32 693	31 741	30 944	30 793	29 885	27 533	22 324
Discounted total lifetime CHD costs^b^ (£)	25 316	22 868	22 639	22 672	22 657	22 619	22 946	22 778	21 646	18 522

^a^Of AMI, ischaemic or haemorrhagic stroke, or fatal CVD.

^b^Discounted at a rate of 3.5% per annum to calculate the present value of the costs.

A patient with SCAD representative of the lowest risk decile (5-year cardiovascular risk of 3.5% and a life expectancy of 26.8 years) would have expected undiscounted costs of £9335 over 5 years, of which 44.7 and 56.8% would be CHD and CVD related, respectively; and undiscounted lifetime costs of £116 888, of which 40.1 and 61.5% would be CHD and CVD related, respectively. A representative patient of the highest risk decile (5-year cardiovascular risk of 44.2% and a life expectancy of 5.51 years) would have expected undiscounted costs of £23 391 over 5 years, of which 53.0 and 72.9% would be CHD and CVD related, respectively; and lifetime undiscounted costs of £43 020, of which 51.9 and 72.5% would be CHD and CVD related, respectively.

**Figure 3:**
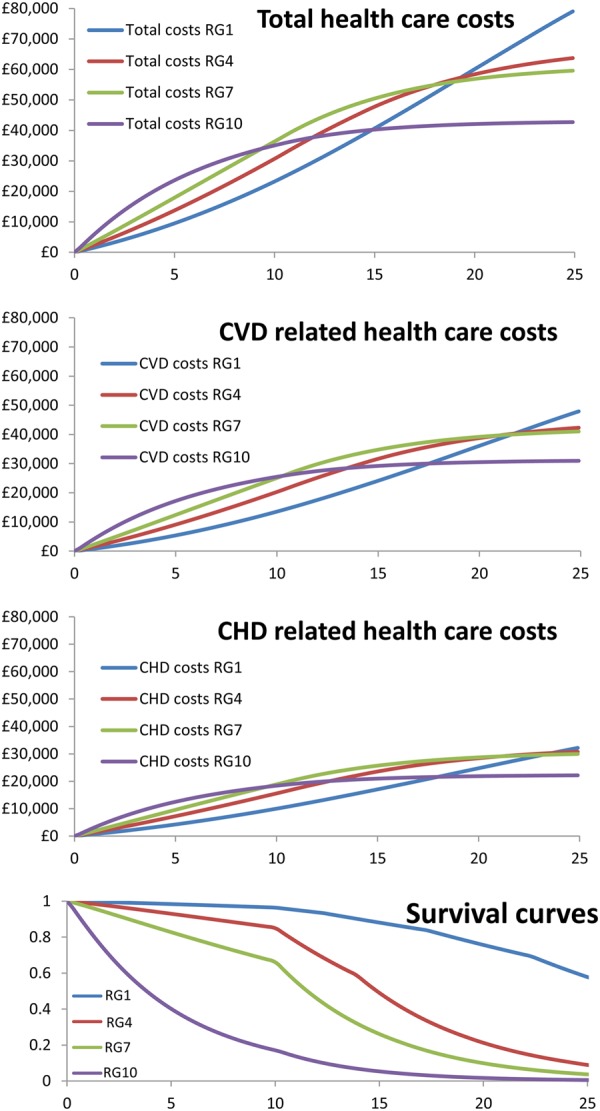
Expected costs and survival over time for patients representative of risk deciles 1, 4, 7, and 10. CHD, coronary heart disease; CVD, cardiovascular disease; RG, risk decile.

*Figure 3* shows the predicted total, CVD-related, and CHD-related costs and the survival curves over a 25-year period for representative patients of risk deciles 1 (lowest risk), 4, 7, and 10 (highest risk). Higher risk patients with SCAD have higher initial costs, which are overtaken by the lower risk patients as a result of higher mortality in the higher risk groups resulting in less time to accrue costs (5-year survivorship differs from 98.4% in risk decile 1 to 40.7% in risk decile 10; and 25-year survivorship differs from 58.2% in risk decile 1 to 0.5% in risk decile 10).

## Discussion

This study addresses a fundamental gap in knowledge relevant to clinicians and policymakers: what is the clinical, health service, and cost burden associated with SCAD? Using data from a large, contemporary, and representative population of patients from the England with SCAD, the analysis has shown that substantial healthcare costs are likely to be incurred as a result of improved ACS survivorship and the ageing population. Moreover, 5-year and lifetime costs vary according to CVD risk, which may be readily predicted from the baseline characteristics of patients that are routinely collected. High-risk patients have considerably higher costs over the initial 5 years but lower lifetime costs than lower risk patients as a result of shorter life expectancy.

Patients with SCAD might be considered to have ‘fallen off the radar’ of clinical interest. Current guidelines give little information about how frequently and where such patients should be followed up or if and how they should be risk stratified.^5,6^ Our results clearly highlight the unmet need and the shortfalls of current approaches—with high use of primary care and frequent hospitalizations, there are considerable ongoing costs. The number of primary care consultations, a mean of 10.8 per patient in the first year in the SCAD cohort, is higher than previous estimates for the overall population (5.5 per year).^22^ In the first year in the SCAD cohort, over a third of patients were hospitalized (and 20.5% for CHD reasons). This is substantially higher than recent estimates for the general population in one area of the UK (14.9%),^23^ and markedly higher than that in the general population for a similar age (23.4 and 21.2% of 60- to 74-year-old males and females, respectively, based on HES data).

Healthcare utilization is, however, insufficient as a metric of the impact on the healthcare system. It is also important to consider the cost imposed on the NHS associated with those resources as this indicates the value of resources that cannot be devoted to health-enhancing activities for other patients. Mean costs of £3133 in the first year in the SCAD cohort are much higher than those in patients without chronic conditions (£293), but comparable with other chronic conditions (e.g. diabetes £3036).^23^ Very high costs in the first year following a non-fatal event during follow-up (MI, ischaemic or haemorrhagic stroke) (£10 377) are reflective of the healthcare utilization required to treat that event.

Patients were stratified by risk to understand the costs accrued in greater detail. This higher resolution analysis allows the identification of where novel treatments and health service interventions have the greatest potential to be cost-effective. Non-CVD co-morbidities were common and had a major influence on costs with e.g. renal disease resulting in a mean extra cost of £1998 per patient in the first year of SCAD. This is an important finding when the presence of multiple co-morbidities has been shown to increase costs^23^ yet clinicians tend to focus only on single diseases.^24^

The panel data analysis examined the average cost per 90-day period with the disease and also the costs of events. The estimated incremental cost of a non-fatal MI over 1 year (£7677 ignoring mortality risk) was lower than some previous estimates from trial populations (e.g. among patients with stable angina the estimate of £9775 from the EUROPA trial).^11^ However, this lower estimate may be reflective of less intensive use of healthcare resources in non-trial settings and should provide a more accurate representation of costs of these events in routine clinical practice. Estimated stroke costs in the first year of the event (£8902 for ischaemic and £10 477 for haemorrhagic stroke incremental to background costs) were similar to those seen in other studies. For example, the OXVASC study estimated mean total healthcare costs per patient in the first year following stroke at £10524.^25^

Furthermore, a study of costs in the first year of SCAD and the first year following an event is of limited use to decision-makers who require more detailed information on the long-term costs and consequences of SCAD. This can be seen from the panel data analysis, which suggested ongoing long-term costs as a result of non-fatal events. By estimating 5-year and lifetime costs by CVD risk group, it was possible to examine the long-term cost implications as a result of the disease and future events. In the shorter term of 5 years, which is shorter than the life expectancy of even the highest risk group (although survivorship in this group was only 40.7% at 5 years), costs increased with cardiovascular risk, and were largely driven by the high number of fatal and non-fatal events among these patients. Over a lifetime, however, patients in the lower risk groups eventually had substantially higher costs than higher risk patients, primarily driven by greater life expectancy and, therefore, costs being incurred over a much longer period. This is a key finding of our research: increased survivorship as well as an increasingly co-morbid and older population will result in significant future healthcare costs. In turn, this has implications for the cost and therefore the cost-effectiveness of established and new SCAD treatments.

Many studies have attempted to address the burden of disease in terms of health losses but fewer have examined the impact on financial costs of disease. Our research used SCAD as an exemplar in estimating resource use and costs from ‘real world’ electronic health record data. The methods used here could be readily applied to other chronic diseases to help produce evidence of their resource and cost implications to better inform clinicians and decision-makers. This would reduce uncertainty for those who need to forecast future care needs and allow for better focused research and development of new technologies and treatments for these chronic diseases as well as resulting in better informed clinical decision-making.

### Limitations

While our study has a number of strengths including the multi-source electronic health record linkage, there are a number of limitations. In addition to being censored at 2010, after which there have been further improvements in the care and survivorship of SCAD, a key weakness of this study was the exclusion of outpatient appointment costs that cannot currently be linked from HES. As a result, the total healthcare costs of this population are underestimated. Further disaggregation of primary care costs into CHD and CVD related was not possible and therefore in each category all primary care costs have been included and therefore are likely overestimated. The estimation of lifetime costs has also involved extrapolation over a longer time period than is currently observed in the CALIBER data. This extrapolation is subject to considerable uncertainty. The SCAD population is also inherently heterogeneous, and there may be value in further disaggregation of the population in future research.

## Conclusions

Using a multi-source electronic health record approach, this study provides, for the first time, estimates and predictors of contemporary national healthcare utilization and costs in the first year of SCAD and the first year following an event. It reveals that patients with SCAD incur substantial healthcare utilization and costs, which vary and may be predicted by 5-year CVD risk profiles. While high-risk patients incur substantially higher costs over the short term (5 years), low-risk patients incur higher lifetime costs as a result of greater life expectancy. Improved cardiovascular survivorship and an ageing UK population will require stratified care in anticipation of the burgeoning economic demand. The methods used here could be readily applied to other chronic diseases to better inform clinical decision-making.

## Authors’ contributions

All authors contributed to the interpretation of the data and several drafts of the manuscript. S.W., M.A., A.M., S.P., and M.S. conducted the statistical analysis and developed the model used to estimate long-term costs. A.D.S., K.A., M.C., C.P.G., A.T., and H.H. provided advice throughout the process. A.T. and H.H. were responsible for the overall grant from the NIHR.

## Appendix

### Model description

A state transition Markov model was used to capture the natural history of SCAD patients. The model predicts time to first non-fatal events (MI, ischaemic stroke or haemorrhagic stroke) and CVD and non-CVD mortality (as a first event or following a non-fatal first event). The model has a lifetime horizon and uses a 90-day cycle length to capture the time varying and age-dependent nature of risks. Risks of the five primary clinical endpoints (non-fatal MI, non-fatal ischaemic stroke, non-fatal haemorrhagic stroke, and CVD and non-CVD mortality) were estimated based on key prognostic factors, including demographic measures, SCAD subtype, use of long-acting nitrates, whether CABG or PCI was performed in the 6 months following CAD diagnosis, previous MI, smoking, blood pressure, diagnosis of hypertension, diabetes, lipids, CVD co-morbidities, non-CVD co-morbidities, psychosocial factors, and clinically assessed biomarkers. Risk equations for the six subsequent events, namely CVD and non-CVD mortality following non-fatal MI, ischaemic stroke, and haemorrhagic stroke were estimated in a similar way. However, due to the greatly reduced numbers of events observed, these were based only on sex and age at time of non-fatal event. Non-CVD mortality beyond the maximum follow-up in the CALIBER data set (10 years) was based on age–sex-specific non-CVD mortality from national life tables. Costs are accrued based on baseline covariate, events experienced, and time alive with the costs based on the results detailed in this article. Full details can be found in Asaria *et al*.^20^

**Table A1:** Patient characteristics

Socio-demographic characteristics	
Sex (% female)	44
Age (if male) (years)	67
Age (if female) (years)	72
Most deprived quintile (%)	20
Baseline diagnoses for entry to the SCAD cohort	
NSTEMI (%)	10
STEMI (%)	7
Unstable angina (%)	14
Stable angina (%)	47
CHD not otherwise specified (%)	23
CVD risk factors	
Current smoker (%)	35
Ex-smoker (%)	32
Never smoked (%)	33
Hypertension (%)	76
Diabetes (%)	16
CVD co-morbidities	
Heart failure (%)	26
Atrial fibrillation (%)	15
Peripheral arterial disease (%)	8
Stroke (%)	9

**Table A2:** Healthcare utilization in first year with stable coronary artery disease and first year following a non-fatal event (myocardial infarction, ischaemic or haemorrhagic stroke)

	Mean (SD)	Median (IQR)
Resource use in first year (*n* = 85 702)		
Hospitalizations		
Hospitalized (%)	37.6 (0.484)	0 (0–1)
Hospitalized for CVD (%)	27 (0.444)	0 (0–1)
Hospitalized for CHD (%)	20.5 (0.403)	0 (0–1)
Inpatient stays	0.875 (4.421)	0 (0–3)
Inpatient stays for CVD	0.503 (2.545)	0 (0–2)
Inpatient stays for CHD	0.343 (1.321)	0 (0–2)
With any hospitalization (*n* = 32 242)		
Inpatient stays	2.325 (6.971)	1 (1–5)
Length of stay	6.717 (17.583)	3 (1–23)
With any hospitalization for CVD (*n* = 23 160)		
Inpatient stays for CVD	1.862 (4.631)	1 (1–4)
Length of stay	7.516 (12.757)	3.667 (1–26)
With any hospitalization for CHD (*n* = 17 532)		
Inpatient stays for CHD	1.674 (2.51)	1 (1–4)
Length of stay	4.579 (7.617)	2 (1–14.75)
Revascularizations		
Any revascularization (%)	5.7 (0.232)	0 (0–1)
PCI (%)	3 (0.17)	0 (0–0)
CABG (%)	2.9 (0.169)	0 (0–0)
Primary care consultations	10.768 (10.857)	8 (0–30)
Drugs		
Patients on any drugs (%)	91.3 (0.281)	1 (0–1)
Patients on CVD drugs (%)	88.2 (0.322)	1 (0–1)
Patients on anticoagulants (%)	8.7 (0.282)	0 (0–1)
Patients on ACEi or ARB (%)	47.7 (0.499)	0 (0–1)
Patients on anti-platelets (%)	65.6 (0.475)	1 (0–1)
Patients on β-blockers (%)	46 (0.498)	0 (0–1)
Patients on calcium channel blockers (%)	31.5 (0.464)	0 (0–1)
Resource in first year following an event (*n* = 6599)		
Hospitalizations		
Hospitalized (%)	83.5 (0.371)	1 (0–1)
Hospitalized for CVD (%)	80.2 (0.399)	1 (0–1)
Hospitalized for CHD (%)	66 (0.474)	1 (0–1)
Inpatient stays	2.364 (6.425)	1 (0–6)
Inpatient stays for CVD	1.931 (5.284)	1 (0–5)
Inpatient stays for CHD	1.434 (3.315)	1 (0–4)
With any hospitalization (*n* = 5512)		
Inpatient stays	2.83 (6.935)	2 (1–6)
Length of stay	14.857 (20.349)	8.5 (1.5–50.5)
With any hospitalization for CVD (*n* = 5291)		
Inpatient stays for CVD	2.408 (5.803)	2 (1–5)
Length of stay	15.541 (20.939)	9 (2–52)
With any hospitalization for CHD (*n* = 4354)		
Inpatient stays for CHD	2.174 (3.88)	1 (1–5)
Length of stay	6.515 (10.857)	3.5 (1–21.5)
Revascularizations		
Any revascularization (%)	13.5 (0.341)	0 (0–1)
PCI (%)	9.1 (0.287)	0 (0–1)
CABG (%)	5 (0.218)	0 (0–1)
Primary care consultations	13.671 (15.407)	11 (0–38)
Drugs		
Patients on any drugs (%)	85.1 (0.356)	1 (0–1)
Patients on CVD drugs (%)	83.6 (0.37)	1 (0–1)
Patients on anticoagulants (%)	14.7 (0.354)	0 (0–1)
Patients on ACEi or ARB (%)	61.5 (0.487)	1 (0–1)
Patients on anti-platelets (%)	76.3 (0.425)	1 (0–1)
Patients on β-blockers (%)	50.1 (0.5)	1 (0–1)
Patients on calcium channel blockers (%)	32.6 (0.469)	0 (0–1)

**Table A3:** Costs in first year with stable coronary artery disease and in first year following an event (myocardial infarction, ischaemic of haemorrhagic stroke)

	Mean (SD) (£)	Median (IQR) (£)
Costs in first year (*n* = 85 702)		
Total costs		
Total cost	3133 (6101)	1149 (43–12 641)
Total CVD cost	2199 (4632)	735 (0–10 274)
Total CHD costs	1780 (3686)	685 (0–8413)
Hospitalizations		
Inpatient costs	2018 (5632)	0 (0–10 603)
Inpatient CVD costs	1487 (4493)	0 (0–9333)
Inpatient CHD costs	1067 (3548)	0 (0–7284)
Primary care costs	466 (463)	361 (0–1291)
Diagnostic test costs	141 (232)	70 (0–538)
Drugs		
All drug costs	508 (1548)	209 (0–1698)
CVD drug costs	105 (113)	84 (0–272)
Costs in first year following an event (*n* = 6599)		
Total costs		
Total cost	10 377 (12 260)	6855 (580–30 755)
Total CVD cost	8916 (10 930)	5819 (384–27 279)
Total CHD costs	6869 (9467)	3972 (159–23 336)
Hospitalizations		
Inpatient costs	8744 (11 554)	5421 (0–27 436)
Inpatient CVD costs	7957 (10 796)	4871 (0–25 843)
Inpatient CHD costs	5910 (9338)	3127 (0–22 020)
Primary care costs	589 (629)	456 (0–1664)
Diagnostic test costs	228 (311)	131 (0–806)
Drugs		
All drug costs	816 (3135)	293 (0–2924)
CVD drug costs	142 (175)	110 (0–376)

**Table A4:** Total healthcare costs per 90-day period

	Coefficient (£)	Standard error	Lower 95% CI (£)	Upper 95% CI (£)
Background cost per 90 days				
Baseline (for a man aged 69 with stable angina and no other co-morbidities)	341	10.15	322	361
Baseline age (centred)	7	0.42	6	7
Female	−7	10.09	−26	13
Increase per 90 days	10	0.18	10	11
SCAD diagnosis (relative to stable angina)				
Other CHD	−20	12.40	−44	5
NSTEMI	157	18.37	121	193
STEMI	−26	21.48	−68	17
Unstable angina	153	14.92	124	183
CVD co-morbidities				
History of heart failure	364	12.23	340	388
History of atrial fibrillation	186	14.32	158	214
History of PAD	327	17.80	292	362
Non-CVD co-morbidities				
Diabetes	338	13.71	312	365
History of liver disease	530	50.70	431	629
History of COPD	231	11.58	208	254
History of cancer	331	17.51	296	365
History of renal disease	756	20.93	715	797
Incremental cost of non-fatal MI				
Cost in first 90-day period	5028	34.41	4961	£5096
Cost in second 90-day period	1282	36.84	1210	£1354
Cost in third 90-day period	675	39.15	598	£751
Cost in fourth 90-day period	692	40.84	612	£772
Cost in subsequent 90-day periods	521	18.64	484	£557
Additional incremental cost of non-fatal MI for patients with diabetes				
Additional cost in first 90-day period	776	75.79	627	924
Cost in second 90-day period	1100	81.56	940	1260
Cost in third 90-day period	785	87.01	614	955
Cost in fourth 90-day period	550	90.87	372	728
Cost in subsequent 90-day periods	369	42.16	286	451
Incremental cost of non-fatal ischaemic stroke				
Cost in first 90-day period	6215	36.01	6144	6285
Cost in second 90-day period	1239	38.03	1164	1313
Cost in third 90-day period	795	41.05	714	875
Cost in fourth 90-day period	654	43.12	569	738
Cost in subsequent 90-day periods	564	19.97	525	604
Incremental cost of non-fatal haemorrhagic stroke				
Cost in first 90-day period	7011	125.41	6765	7257
Cost in second 90-day period	1767	138.80	1495	2039
Cost in third 90-day period	947	155.22	643	1252
Cost in fourth 90-day period	751	165.14	428	1075
Cost in subsequent 90-day periods	927	74.20	782	1073
Cost of fatal events				
Fatal CVD event	2008	24.88	1960	2057
Fatal non-CVD event	2240	20.16	2201	2280

**Table A5:** Total cardiovascular disease-related healthcare costs

	Coefficient (£)	Standard error	Lower 95% CI (£)	Upper 95% CI (£)
Background cost per 90 days				
Baseline (for a man aged 69 with stable angina and no other co-morbidities)	224	7.74	209	239
Baseline age (centred)	6	0.32	6	7
Female	−23	7.65	−38	−8
Increase per 90 days	7	0.15	6	7
SCAD diagnosis (relative to stable angina)				
Other CHD	2	9.39	−16	21
NSTEMI	145	13.66	119	172
STEMI	29	15.77	−2	60
Unstable angina	125	11.32	103	147
CVD co-morbidities				
History of heart failure	248	9.29	230	266
History of atrial fibrillation	221	10.90	199	242
History of PAD	242	13.53	216	269
Non-CVD co-morbidities				
Diabetes	194	10.43	173	214
History of liver disease	279	38.68	203	355
History of COPD	142	8.79	124	159
History of cancer	154	13.34	128	180
History of renal disease	418	16.07	386	449
Incremental cost of non-fatal MI				
Cost in first 90-day period	4854	29.55	4796	4911
Cost in second 90-day period	1209	31.64	1147	1271
Cost in third 90-day period	640	33.63	574	706
Cost in fourth 90-day period	675	35.08	606	744
Cost in subsequent 90-day periods	481	15.64	451	512
Additional incremental cost of non-fatal MI for patients with diabetes				
Additional cost in first 90-day period	674	65.05	546	801
Cost in second 90-day period	1042	70.02	904	1179
Cost in third 90-day period	660	74.71	514	807
Cost in fourth 90-day period	403	78.04	250	556
Cost in subsequent 90-day periods	280	35.33	210	349
Incremental cost of non-fatal ischaemic stroke				
Cost in first 90-day period	5957	30.95	5897	6018
Cost in second 90-day period	1151	32.69	1087	1215
Cost in third 90-day period	675	35.29	606	744
Cost in fourth 90-day period	539	37.08	466	612
Cost in subsequent 90-day periods	448	16.86	415	481
Incremental cost of non-fatal haemorrhagic stroke				
Cost in first 90-day period	6836	107.73	6625	7047
Cost in second 90-day period	1517	119.28	1284	1751
Cost in third 90-day period	585	133.45	324	847
Cost in fourth 90-day period	393	142.02	115	671
Cost in subsequent 90-day periods	670	62.69	547	792
Cost of fatal events				
Fatal CVD event	2071	21.30	2029	2113
Fatal non-CVD event	1737	17.28	1703	1771

**Table A6:** Total coronary heart disease-related healthcare costs

	Coefficient (£)	Standard error	Lower 95% CI (£)	Upper 95% CI (£)
Background cost per 90 days				
Baseline (for a man aged 69 with stable angina and no other co-morbidities)	179	5.93	167	191
Baseline age (centred)	4	0.24	4	5
Female	−23	5.85	−35	−12
Increase per 90 days	4	0.12	3	4
SCAD diagnosis (relative to stable angina)				
Other CHD	82	7.17	68	97
NSTEMI	219	10.49	198	239
STEMI	111	12.04	88	135
Unstable angina	163	8.65	146	179
CVD co-morbidities				
History of heart failure	143	7.11	129	157
History of atrial fibrillation	84	8.34	67	100
History of PAD	161	10.35	141	181
Non-CVD co-morbidities				
Diabetes	144	7.98	128	160
History of liver disease	198	29.62	140	256
History of COPD	117	6.72	104	131
History of cancer	107	10.21	87	127
History of renal disease	201	12.34	176	225
Incremental cost of non-fatal MI				
Cost in first 90-day period	4658	23.69	4612	4705
Cost in second 90-day period	1166	25.36	1116	1215
Cost in third 90-day period	590	26.96	538	643
Cost in fourth 90-day period	642	28.13	587	697
Cost in subsequent 90-day periods	475	12.40	451	500
Additional incremental cost of non-fatal MI for patients with dibetes				
Additional cost in first 90-day period	643	52.14	541	745
Cost in second 90-day period	792	56.13	682	902
Cost in third 90-day period	701	59.90	584	819
Cost in fourth 90-day period	330	62.57	207	453
Cost in subsequent 90-day periods	269	28.01	215	324
Incremental cost of non-fatal ischaemic stroke				
Cost in first 90-day period	3029	24.82	2981	3078
Cost in second 90-day period	620	26.22	568	671
Cost in third 90-day period	415	28.31	359	470
Cost in fourth 90-day period	262	29.74	203	320
Cost in subsequent 90-day periods	256	13.42	230	282
Incremental cost of non-fatal haemorrhagic stroke				
Cost in first 90-day period	2874	86.38	2704	3043
Cost in second 90-day period	790	95.66	602	977
Cost in third 90-day period	218	107.04	8	428
Cost in fourth 90-day period	301	113.92	77	524
Cost in subsequent 90-day periods	251	49.89	154	349
Cost of fatal events				
Fatal CVD event	1407	17.06	1374	1441
Fatal non-CVD event	1068	13.84	1040	1095

**Table A7:** Covariate profiles based on deciles of 5-year risk

Patient average covariate profiles based on deciles of 5-year risk of composite CVD first event										
Risk decile	1	2	3	4	5	6	7	8	9	10
5-year risk (average)	3.69%	5.70%	7.37%	9.15%	11.20%	13.71%	17.14%	22.14%	30.42%	52.37%
Re-calculated 5-year risk	3.46%	5.43%	6.95%	8.53%	10.36%	12.57%	15.64%	20.07%	27.23%	44.18%
Socio-demographic characteristics										
Sex (female)	64%	48%	42%	39%	37%	37%	38%	42%	44%	46%
Age (if male)	49	55	59	62	65	67	71	74	77	81
Age (if female)	53	62	67	70	73	75	78	80	83	87
Age (weighted average)	52	59	62	65	68	70	73	76	80	84
Most deprived quintile	15%	17%	18%	19%	20%	21%	21%	22%	22%	24%
SCAD diagnosis and severity										
Other CHD	11%	17%	20%	22%	24%	24%	25%	26%	25%	20%
NSTEMI	0%	1%	3%	5%	8%	10%	12%	17%	23%	43%
STEMI	1%	4%	8%	12%	13%	14%	13%	9%	6%	4%
Unstable angina	10%	13%	12%	12%	12%	12%	13%	15%	17%	15%
Stable angina	78%	65%	56%	49%	43%	39%	37%	34%	29%	18%
PCI in last 6 months	9%	12%	13%	14%	13%	13%	11%	9%	6%	4%
CABG in last 6 months	9%	7%	6%	5%	5%	4%	4%	3%	2%	1%
Previous/recurrent MI	2%	6%	10%	14%	18%	23%	26%	29%	32%	43%
Use of nitrates	10%	16%	19%	21%	24%	28%	33%	37%	43%	56%
CVD risk factors										
Current smoker	31%	35%	36%	37%	38%	38%	37%	35%	32%	30%
Ex-smoker	27%	30%	31%	32%	32%	33%	34%	34%	34%	34%
Never smoked	41%	35%	33%	31%	30%	29%	29%	31%	33%	36%
Hypertension	69%	70%	71%	71%	72%	74%	76%	79%	83%	87%
Diabetes	4%	8%	10%	12%	14%	16%	18%	21%	24%	32%
Total cholesterol (mmol/L)	4.95	4.91	4.84	4.79	4.74	4.74	4.70	4.68	4.64	4.54
HDL (mmol/L)	1.41	1.37	1.35	1.35	1.35	1.35	1.36	1.37	1.37	1.35
CVD co-morbidities										
Heart failure	5%	7%	9%	12%	15%	19%	27%	37%	52%	73%
Peripheral arterial disease	1%	2%	3%	4%	6%	8%	10%	13%	16%	25%
Atrial fibrillation	3%	5%	7%	9%	10%	13%	16%	21%	29%	43%
Stroke	0%	1%	1%	2%	3%	5%	8%	14%	22%	39%
Non-CVD co-morbidities										
Chronic kidney disease	2%	2%	3%	4%	4%	5%	7%	9%	12%	20%
Chronic obstructive pulmonary disease	20%	20%	20%	21%	22%	23%	25%	27%	28%	30%
Cancer	4%	5%	6%	7%	8%	9%	11%	13%	14%	12%
Chronic liver disease	0%	1%	1%	1%	1%	1%	1%	1%	1%	1%
Psychosocial characteristics										
Depression at diagnosis	20%	17%	15%	15%	14%	14%	15%	17%	18%	21%
Anxiety at diagnosis	7%	6%	6%	7%	7%	7%	8%	8%	10%	12%
Biomarkers										
Heart rate (b.p.m.)	72	71	71	71	71	71	72	73	74	76
Creatinine (mmol/L)	88	92	95	96	98	100	101	104	109	125
White cell count (10^9^/L)	6.81	7.05	7.19	7.31	7.44	7.54	7.62	7.76	7.88	8.22
Haemoglobin (g/100 mL)	14.26	14.26	14.16	14.05	13.88	13.70	13.48	13.16	12.81	12.20
